# Some Comments on the Methods of To-Day

**Published:** 1920-10-16

**Authors:** 


### SYPHILIS AND ITS TREATMENT.

### Some Comments on the Methods of To-day.

It is but a very short time ago that a large series of treatment schemes for the major venereal disease was paraded before the profession's notice. Now, aided by the opportunities which military organisation brought, it has become gradually accepted that we can in practice condense the whole into one or other of certain routine "courses" administered at the various clinics throughout the country. And, critically considered, there appears to be but one underlying principle employed. We are treating the Wasserman reaction.

But are we, meanwhile, giving sufficient attention to the unfortunate individual himself? In the organised treatment of most other diseases our methods should be, and usually are, based on knowledge of the patient's general health, his hereditary disease tendencies, a study of his habits, his occupations, and his total physical condition. In all medical teaching this principle has been considered as axiomatic, and yet it seems that we are to-day tending more and more towards failure to recognise it when dealing with syphilitic infections. Whatever the *diagnostic* value of the serological tests—and it has been conclusively proved as a high one—there is little doubt that occasionally a negative Wasserman may be found to occur during active manifestations of the disease, and certainly positives may be recorded in a syphilitic in whom some obvious and active pathological lesion exists, but from a cause quite other than spirochætal infection. In either case, the misleading influence of the laboratory test is obvious. Yet it is impossible to deny the fact that with the great majority of practitioners the present-day objective is the assumedly certain "cure" to be obtained by specific chemical means.

### A World-wide Tendency.

Recently the American *Medical Record* published an account of the parallel situation developing in the U.S.A., and, difficult as it is to believe that this criticism is universally applicable, yet if such is indeed becoming the world-wide conception of one of the most brilliant and most valuable therapeutic triumphs, then it is undoubtedly time that the medical profession paused to reflect upon the real behaviour of this most insidious of all diseases.

Our American colleague, Dr. W. W. Graves of St. Louis University, asks three very serious questions: In the first place, have we any justification for the present-day teachings and practices in the treatment of syphilis? In the second, are our brilliant so-called "cures" new, or have they ever been, with but few exceptions, other than symptomatic cures? And thirdly, have we any biological or clinical evidence justifying the conclusion that the favourable influence of remedies in human syphilis is dependent upon any other factor than their influences in the defensive mechanism inherent in the syphilitic?

On this side of the Atlantic a number of medical men will, in reply, instance the published records of non-recurrence in a considerable number of the cases whose treatment was commenced under Service conditions and in Service establishments during the early part of the war. But it is unnecessary to point out, concerning these deductions, the limitations which are imposed both by the circumstances and the short time over which they extend. It is essential to take the broader view, and, above all, to remember the true pathology and the natural history of the syphilitic infection. If those be considered critically we find that there is relatively good agreement concerning the earlier constitutional manifestations of the disease and the reaction of the whole organism to spirochætal invasion which the initial phases represent. But as these early symptoms subside, either of their own accord or after one of the accepted "cures," a very great number of patients enter into the *latent periods* of syphilis.

### Latent Syphilis.

Now, it is concerning these latent periods that a large element of doubt still exists. Sometimes they develop very early, and sometimes, perhaps fortunately, not at all. In any case, investigation into the clinical history of syphilitics who do pass into the latent condition must leave a clinician with but little doubt that therein many minor breaks in health occur which should strictly be interpreted as manifestations of syphilis, although they are sufficiently indefinite to escape classification as such. As Dr. Graves remarks, scarcely any organ or tissue may remain exempt throughout the so-called latent periods of the infection, and it must be admitted that experience so far suggests that frank manifestations of the disease may occur or recur at any time in the life of the individual, however thoroughly we may have treated him during the earlier days of his trouble. Also, if undue reliance be placed on laboratory tests and routine courses, nothing is more likely than that cardio-vascular changes, alterations in reflexes, in the special senses, pupils, pallor and pigmentation may develop insidiously and unobserved by doctor and patient alike. Many of the "cured" individuals certainly present a markedly lowered general resistance, and, with Dr. Graves, one is forced to agree that the greater part of clinical evidence is in support of the idea that the virus, once it has invaded the tissues of the body, so long as it remains, seldom if ever becomes wholly and completely dormant.

No doubt intravenous arsenic administration has, in a large number of successful cases, completely killed off the invading organism. These may form a large or small proportion of the whole. At present there are insufficient reliable figures from which to form a judgment, but it will be surprising if there is not a large residue of cases which have entered, in spite of their negative Wasserman re-

**Syphilis and its Treatment**—(*continued*).

actions, the latent and not the cured phase. The truth of the position appears to be that no one can as yet say exactly when a syphilitic is cured—cured, that is, in the sense that his system is absolutely free from the virus. Even if this seems, relative to the popular beliefs of the day, a too pessimistic statement, yet it is probably true. There is, indeed, little finality in our position with regard to this branch of venereal treatment. The various methods now employed are, compared with those of even twenty years ago, remarkable for their power and rapidity of action; but we have still more steps of the ladder to climb, and these particularly concern the efficient clinical observation and control of the latent periods. Here there is a field calling urgently for the fullest investigation. It is certain that this need and the therapeutic limitations which give rise to it are realised by the leaders of medical research; but there are two spheres of anti-venereal activity in which the true facts must also be thoroughly appreciated. First, there is the great army of general practitioners now actively treating syphilis by the so-called "specific" methods, and, secondly, the large number of well-intentioned laymen who are taking active interest in the rapid extension of venereal treatment centres. Repeatedly has the paramount value of prevention been urged. The essential limitations of specific treatment form a section of the venereal problem calling no less urgently for general appreciation.

